# Policy and Behavior: Comparisons between Twitter Discussions about the US Tobacco 21 Law and Other Age-Related Behaviors

**DOI:** 10.3390/ijerph19052613

**Published:** 2022-02-24

**Authors:** Page D. Dobbs, Jason B. Colditz, Shelby Shields, Anna Meadows, Brian A. Primack

**Affiliations:** 1 Health, Human Performance and Recreation Department, University of Arkansas, Fayetteville, AR 72701, USA; smshield@uark.edu (S.S.); aemeadow@uark.edu (A.M.); 2 Center for Research on Media, Technology and Health, University of Pittsburgh School of Medicine, Pittsburgh, PA 15213, USA; colditzjb@pitt.edu; 3 College of Education and Health Professions, University of Arkansas, Fayetteville, AR 72701, USA; bprimack@uark.edu; 4 College of Public Health and Human Science, Oregon State University, Corvallis, OR 97331, USA

**Keywords:** Tobacco 21, tobacco use, health communication, sentiment, age-restriction

## Abstract

To combat the e-cigarette epidemic among young audiences, a federal law was passed in the US that raised the minimum legal sales age of tobacco to 21 years (commonly known as Tobacco 21). Little is known about sentiment toward this law. Thus, the purpose of our study was to systematically explore trends about Tobacco 21 discussions and comparisons to other age-restriction behaviors on Twitter. Twitter data (n = 4628) were collected from September to December of 2019 that were related to Tobacco 21. A random subsample of identified tweets was used to develop a codebook. Two trained coders independently coded all data, with strong inter-rater reliability (κ = 0.71 to 0.93) found for all content categories. Associations between sentiment and content categories were calculated using χ^2^ analyses. Among relevant tweets (n = 955), the most common theme—the disjunction between ages for military enlistment and tobacco use—was found in 17.8% of all tweets. Anti-policy sentiment was strongly associated with the age of military enlistment, alcohol, voting, and adulthood (*p* < 0.001 for all). Opposition to Tobacco 21 propagates on social media because the US federal law does not exempt military members. However, the e-cigarette epidemic may have fueled some support for this law.

## 1. Introduction

Between June 2019 and February 2020, the US faced an e-cigarette and vaping-related illnesses (EVALI) epidemic that caused 2807 hospitalizations and 68 deaths in the US, with 52% of EVALI cases found among those less than 24 years of age [1]. This health crisis led members of the public health community to urge the government and governmental agencies (e.g., FDA) to pass meaningful policies to reduce youth and young adult access to and use of all tobacco products. Therefore, on 20 December 2019 the US federal government enacted “Tobacco 21”, which raised the minimum legal sales age (MLSA) of tobacco (which includes e-cigarettes) to 21 years [2], a law that several other countries are now considering [3]. In the US, this law levies penalties for retailers and/or retail employees who violate it by selling tobacco products to those under 21 [4]. With evidence of Tobacco 21’s efficacy to reduce tobacco initiation and use [2,5,6,7], the federal law was met with bipartisan support and was expected to reduce tobacco (including e-cigarette) use among youth and young adults [2].

Tobacco sales have been historically restricted by age in the US; the first minimum age of legal access for tobacco products was set to 16 years of age in New Jersey in 1883 [8]. Tobacco industry marketing documents indicate that young consumers are essential for business [8], and thus traditional tobacco campaigns have targeted particular young populations, including the US military community [9,10,11]. Most new military recruits are between 18 and 21 years, which are known to be particularly vulnerable ages for tobacco initiation [12]. Furthermore, research has found that 41% of a military sample reported that they initiated smoking during their time in the military [13]. Recently, e-cigarette companies such as JUUL have mimicked these marketing strategies by creating military focused campaigns [14]. With as many as 20% and 30% of active military and veterans reporting that they use e-cigarette products [15,16] and combustible cigarettes [17], respectively, it is not surprising that the tobacco industry aggressively supports military exemptions from Tobacco 21 laws [14]. These exemptions are concerning due to evidence that e-cigarette use among nicotine-naïve youth and young adults increases their risk for initiation and prolonged use of combustible cigarettes [18,19,20] which could translate to higher morbidity and mortality rates among military members’ as well as increases in the Veterans Health Association health care costs [4].

The federal law closed the gap in Tobacco 21 policies throughout the 50 US states [21], and it did not exempt military members; thus, making it illegal to sell tobacco products (including e-cigarettes) to all people under 21 years, including military members. This sudden policy change—especially as it relates to the military—raises critical questions for optimal implementation and enforcement of the new law, especially given its difference from other age-restricted behaviors (e.g., MSLA for alcohol and legal voting age). Understanding public perception of laws dramatically increases the likelihood of avoiding potential pitfalls, facilitating enforcement, and amending laws as necessary [22,23,24,25]. Prior to enactment, polling studies suggested that there was broad public support for Tobacco 21 policies in the US [22,26,27,28,29]. However, other studies about Tobacco 21 laws have highlighted public opposition, such as protests against “the nanny state” [30], a reference that implies that a government is overprotective of its citizens. Still others have claimed that raising the MLSA of tobacco products for military members violates the Equal Protection Clause of the US Constitution, a claim normally not supported by courts [29]. However, none of these studies were conducted during the height of the EVALI outbreak.

An important gap in the literature is that little is known about public perception of Tobacco 21 from organic sources such as social media, which is highly utilized by young audiences; 97% of those under 18 years reported using at least one social media channel in 2018 [31]. To date, research about tobacco use discussions on social media have focused on addiction and use among young audiences [32,33]; however, there are limited social media-based studies regarding tobacco control policies, particularly federal policies [34,35]. Social media data provides valuable organic information via a public platform that can explore conversations about emerging tobacco products [32,33,36,37] and public response to tobacco control policies [34,35,38] that would take much longer to access if using traditional methodologies (e.g., polling surveys). Thus, it may be illuminating to systematically study information from social media in this way to determine meaningful steps moving forward that may help optimize implementation, enforcement, and potential amendment of the Tobacco 21 law. Further, given the availability for public social dialog on Twitter and its utilization in past tobacco control research [34,35,38], it provides an optimum platform for policy-based discussions. Therefore, the purpose of our exploratory study was to systematically examine trends about Tobacco 21 policy discussions on Twitter. More specifically, we sought to explore if there was a significant relationship between sentiment (pro, anti, neutral) about Tobacco 21 and discussions about the type of tobacco mentioned and other age-related behaviors, such as enrollment in the military.

## 2. Methods

Data for the current study were collected via publicly available “RITHM” software [39], which we used to retrieve real-time Twitter messages (i.e., tweets) occurring between 1 September 2019 and 31 December 2019, the months immediately before and the days after the federal law was enacted and during the height of the EVALI outbreak. Tweets were captured using keyword search terms to identify content related to the Tobacco 21 law. Primary search terms were related to tobacco and nicotine products (e.g., tobacco, cigarette, e-cigarette, vaping) and were further filtered with terms specific to Tobacco 21 (i.e., 18, 21, age, buy, mcconnell, mitch, purchase, t21, tobacco21). Because the first Tobacco 21 bill presented to the US Senate was championed by Senator Mitch McConnell (Republican, Kentucky, Senate Majority Leader in 2019), his name appeared in news and social media posts about this topic and was included as a search term. Keywords were inclusive of hashtags (i.e., “#” prefix on text) and capitalization (e.g., “t21” also captured “#T21”). Using these search filters, we identified 615,574 tweets, which were reduced to 231,447 tweets after removing redundant “retweets” (i.e., sharing others’ tweets). For feasibility of qualitative annotation, a random subsample of 2% of tweets resulted in a dataset of 4628 tweets for human coding. Similar sample sizes and sampling procedures have provided adequate representation of Tweets in prior Twitter-based research [33].

Two trained coders (SS & AM) were provided with a structured data collection form that included the original tweet text, text of the quoted tweet if the original tweet contained another embedded tweet within it, and a link to the online version of each tweet. Coders were permitted to click the link and further review the tweet on Twitter for added context such as embedded images or videos. All tweets were first coded for relevance to Tobacco 21 and then relevance to the Federal Tobacco 21 policy (e.g., did not mention a state or locality). Tweets that mentioned a state or local policy—and not the federal policy of interest—were excluded from analysis.

### 2.1. Qualitative Codebook Development

Development of the codebook followed Crabtree and Miller’s procedures established for public health qualitative research [40]. Three researchers first examined 200 tweets using in-vivo coding to search for nuanced information related to topics such as sentiment and relevance to Tobacco 21 laws. Using this inductive coding procedure [41], descriptive codes about Tobacco 21-related topics were identified. After the researchers met to compare initial coding, they employed a grounded theory approach to hone the codebook by adding, splitting, expanding, deleting, and/or collapsing codes. To provide transparent and relevant direction for coding, each code was operationally defined, and examples and concise definitions were developed for each code (see Table 1).

### 2.2. Coding Scheme

The final codebook included 15 codes (capitalized) that represented four categories: (1) relevance, (2) sentiment, (3) type of tobacco, and (4) age-restricted behaviors. 

Relevance. Relevance determined whether the tweet was about a TOBACCO 21 bill/law, and if the tweet did indeed refer to the FEDERAL TOBACCO 21 law. Tweets that mentioned a state or locality were excluded from the study.

Sentiment. Each tweet was categorized as expressing support for the Tobacco 21 policy (PRO-POLICY), opposing the policy (ANTI-POLICY), or neither (NEUTRAL-POLICY).

Type of Tobacco. The type of tobacco mentioned in the tweet was coded if it included a type of tobacco that was restricted. Four codes were used to indicate CIGARETTES OR CIGARILLOS, E-CIGARETTES OR VAPOR PRODUCTS, NICOTINE, and OTHER (i.e., dip, cigars, hookah). Some tweets mentioned more than one type of tobacco in a single tweet. 

Age-Restricted Behaviors. Codes related to age-restricted behaviors included ENLISTMENT IN THE MILITARY, VOTING, and the right to PURCHASE A FIREARM. Age-restricted behaviors also included the use or purchase of ALCOHOL or CANNABIS. Discussions about age-restriction also discussed the age that constituted adulthood which was coded as ADULTHOOD.

### 2.3. Coding Procedures

Next, two independent coders were trained via a two-hour training that included a review of the literature, conceptual background related to the coding process, precise definitions of coding terms, and hands-on practice with sample Tweets. Next, coders independently coded 500 tweets per round to allow checks for reliability, clarification of operational definitions, and further discussion about the codebook. Using Cohen’s κ to measure inter-rater reliability [42], acceptable agreement, defined *a priori* as κ > 0.60 as defined by established metrics [43] was achieved after the first round of coding (κ = 0.71 to 0.93). All discrepancies between codes were then adjudicated to provide final data. This process began with the two coders meeting to discuss all discrepancies. On the rare occasions when they were unable to reach agreement (<1%), the Principal Investigator (PDD) provided adjudication.

### 2.4. Analysis

Grounded theory was used to systematically create relevant codes (via in vivo coding) to organize the data into categories that captured its full richness [44]. After coding 4628 tweets, those not relevant to Tobacco 21 were removed and frequencies for codes were assessed using a quasi-statistical qualitative methodology [40]. We summed counts for each code and then computed statistical relationships between the coded sentiment (pro, anti, neutral) and the other content categories using χ^2^ analyses with a two-tailed alpha set *a priori* to 0.05.

## 3. Results

Overall, 955 (21.6%) of the 4628 tweets were identified as relevant to the federal Tobacco 21 law. Among these 955 messages, 405 (42.4%) opposed Tobacco 21, 143 (15.0%) supported the law, and 407 (42.6%) were neither supportive of nor opposed to the law. Associations between sentiment and other codes related to the federal Tobacco 21 law (i.e., type of tobacco and age-related discussions) are presented in Table 2.

### 3.1. Type of Tobacco

Of the 955 tweets that discussed the federal Tobacco 21 law, 402 (42.1%) mentioned a specific type of tobacco affected by the law: 227 (23.8%) stated that the law restricted access to cigarettes or cigarillos, 193 (20.2%) to e-cigarettes or vapor products, and 46 (4.8%) to nicotine (e.g., “*So, @FDATobacco is T-21 now the law? If someone who is 20 years old walks into a store and wants to buy a bottle of nicotine eliquid, can it legally be sold to them?*”). Some tweets, such as this one, mentioned that the law restricted access to more than one product: “*Did he tell them T21 took away their rights to smoke or vape? # WeVapeWeVote*”. Of those that mentioned an age restriction to cigarettes, 52.9% were associated with anti-policy sentiment (χ^2^ [1, N = 955] = 13.3, *p* < 0.001) and 33.5% with neutral policy sentiment (χ^2^ [1, N = 955] = 10.2, *p* < 0.001), see Table 2. Among tweets that mentioned restriction to e-cigarettes and vapor products, 21.8% were associated with pro-policy sentiment (χ^2^ [1, N = 955] = 8.8, *p* < 0.01), 28.5% with anti-policy sentiment (χ^2^ [1, N = 955] = 19.2, *p* < 0.001), and 49.7% with neutral policy sentiment (χ^2^ [1, N = 955] = 5.0, *p* < 0.05).

### 3.2. Age-Related Behaviors

Most (82.4%) of the 170 tweets that compared military enlistment and Tobacco 21 were associated with anti-policy sentiment (χ^2^ [1, N = 955] = 135.1, *p* < 0.001); 8.8% of these tweets endorsed pro-policy sentiment (χ^2^ [1, N = 955] = 6.2, *p* < 0.05) and 8.8% endorsed neutral sentiment toward the law (χ^2^ [1, N = 955] = 96.6, *p* < 0.001). Some included strong disapproval by directly referring to the policy initiative as “stupid” or “dumb”, while others made sarcastic comments such as “*This makes sense*” and “*This country is absolutely hilarious at times. Sorry lads, no smokes for you but you can go die at war for us:)*”.

There were also associations between policy sentiment and comparisons with other age-related behaviors. Of tweets that mentioned buying/selling alcohol (n = 103), 67% were associated with anti-policy sentiment (χ^2^ [1, N = 955] = 28.6, *p* < 0.001) and 16.5% with neutral policy sentiment (χ^2^ [1, N = 955] = 32.2, *p* < 0.001). For example, one anti-policy tweet responded to enactment of the law as follows: “*But an 18 yr old American man and woman can join The Army Navy AirForce Nt’l Guard. Fight and Die for their country that won’t let them buy cigarettes or alcoholic beverages. What kind of sense does that make?*” Of tweets that compared the law to the legal voting age (n = 57), 73.7% were associated with anti-policy sentiment (χ^2^ [1, N = 955] = 24.3, *p* < 0.001) and 8.8% with neutral-policy sentiment (χ^2^ [1, N = 955] = 28.4, *p* < 0.001). Some tweets focused on other political topics, including gun ownership (n = 36, 3.8%); 63.9% and 13.9% were associated with anti-policy (χ^2^ [1, N = 955] = 7.1, *p* < 0.01) and neutral-policy (χ^2^ [1, N = 955] = 12.6, *p* < 0.001) sentiment, respectively. For example, one tweet read, “*I just think it’s funny I can legally go buy a gun right now but I can’t buy tobacco wow that’s crazy*”. Lastly, 35 (3.7%) of the tweets discussed the age that constituted adulthood. Among these, 85.7% were associated with anti-policy sentiment (χ^2^ [1, N = 955] = 27.9, *p* < 0.001) and 5.7% were associated with neutral-policy sentiment (χ^2^ [1, N = 955] = 20.2, *p* < 0.001). For example, one Twitter user stated, “*Non-smoker here, don’t even see the appeal, but I heavily dislike the idea of raising the legal age of things above the “legally adult” line. Either an 18-year-old is an adult, or they aren’t*”.

## 4. Conclusions

We sought to systematically explore trends about Tobacco 21 and age-related behaviors on Twitter by exploring the relationship between sentiment toward the policy and discussions about the type of tobacco mentioned and other age-related behaviors. Given that past research has highlighted public support for Tobacco 21 policies [22,26,27,28,29], our study adds unique findings about public opposition from Twitter users prior to enactment of the federal law. Our findings highlight similar policy opposition identified by others, such as protests against a “nanny state” and suggestions that those who are willing to enlist in the military should be able to use tobacco products [30]. Such sentiment provides context to the political climate at the time the federal Tobacco 21 policy was passed, which was influenced by supportive “front groups” such as vape shop owners, employees, and advocacy organizations that were provided resources by the tobacco industry to appear as a “grassroots” network [45]. These findings are important for other countries that are considering policies to raise their tobacco sales age. Social media platforms may foster negative chatter about the policy or policy components, particularly around the time of enactment; however, tobacco prevention agencies and government enforcement agencies can attempt to control the narrative spread on social media by using these resources to educate the public. Social media have been historically used to set agendas of news coverage [46], particularly about e-cigarettes [47], due to its capability of providing information instantaneously and its ability to generate discussion among users not necessarily connected (e.g., non-followers) with a social network. Thus, those implementing tobacco control policies should distribute information about tobacco control policies on social media platforms via public health networks (e.g., prevention agencies) to garner public support, which can help with policy adoption and smooth implementation.

Although the Family Smoking Prevention and Tobacco Control Act of 2009 gave the US Food and Drug Administration (FDA) regulatory authority of tobacco products (e.g., oversight of compliance checks of retailers) [48], it also established that only Congress could change the federal MLSA of tobacco products [2]. This provision made it more difficult to raise the MLSA than it would have been if the FDA was simply provided this administrative authority. Despite this regulatory hurdle, pressure was placed on the FDA, US Congress, and the president to enact meaningful policies that would reduce youth and young adult access to e-cigarettes during the EVALI outbreak (from June 2019 and February 2020). Among policy supportive tweets identified in our study (n = 143, 15.0%), 42 (29.4%) mentioned e-cigarettes. Some of these tweets included pleas from the vaping community that expressed support for the Tobacco 21 law as long as all vapor products were not banned entirely. Thus, public outcry for regulatory responses to the EVALI outbreak may have fueled support for Tobacco 21 from unlikely sources, such as the vaping community, because they saw it as sensible policy compared to more drastic approaches. 

Following the Master Tobacco Settlement agreement, the tobacco industry was required to surrender internal marketing documents. These documents described in vivid detail the strategies used by the tobacco industry to target youth and young adults [49]. They also revealed the industry’s fear that age-restriction laws (i.e., raising the MLSA to 21 years) may substantially impair their business model [50]. Interestingly, the leading e-cigarette brand from 2018–2019, JUUL, publicly supported local- and state-level Tobacco 21 laws [51]. Such policy support from JUUL appeared to promote their social responsibility, similar to social media messages depicted by transnational tobacco companies [35]. However, some cautioned that JUUL, which was partially owned by Altria (the tobacco conglomerate that owns Phillip Morris USA and the Marlboro cigarette brand) may have supported Tobacco 21 policies that included negative components (e.g., military exemptions) [4], using similar tactics of legislative influence as those used in smoke-free air laws passed before 2000 [52].

Although the federal law does not include a military exemption, it is important to acknowledge public opinion and policy discussions about health-related policies. This information may influence implementation and enforcement of tobacco control laws. Given that 17.8% of tweets about Tobacco 21 mentioned the military, and 82.4% of tweets about the military used anti-policy sentiment, our findings may have practical implications. Based on our findings, the federal policy may not have been well received by the military community (including those who simply support the military and are not necessarily military personnel or dependents). Thus, in order to ensure compliance with the federal law, enforcement agencies should make sure that compliance checks are conducted for all retailers within and around military communities. Recent evidence indicates there may be an excess number of tobacco retailers and vape shops surrounding military bases (i.e., Fort Bragg in North Carolina) [53]; thus, research is needed to determine if tobacco retailers in these areas are appropriately enforcing the Tobacco 21 law among military personnel.

Furthermore, our findings suggest a need to explore the origins of public opinions about Tobacco 21. Tweets made by vape shop owners may reflect a larger political message shared by the tobacco industry. While tobacco companies have made political position statements about tobacco control policies on Twitter [35], they may also promote their message via other less obvious approaches. Modern tobacco and e-cigarette companies have evolved from using their employees as the face of political movements to funding front groups to advance the industry’s message [45]. However, promotion of industry-wide political interests about recently enacted youth access policies has been insufficiently explored. Future research should examine tobacco and e-cigarette companies’ diffusion of information about tobacco control policies via social media networks—including front groups’ activities such as local vape shops and advocacy groups. Researchers, public health advocates, and policymakers could use such data to better understand how the tobacco industry influences public opinion.

Moreover, some tweets within the military category appeared to describe the origins of opinions about Tobacco 21. These messages may be created by the tobacco industry and may reflect industry marketing rather than the view of the general public or the broader US military community [10,11,14]. For example, one user stated: “*When I worked on Tobacco 21 during law school, not something I personally support, military commanders were advocates for the bill because it means their soldiers are in better health and more effective*”. The general public may believe they are supporting the interest of the military by supporting military exemptions, when in fact they are perpetuating the tobacco industry’s marketing messages [14].

### Limitations

Although Twitter provides a public platform for opinion and discussion, it does not represent the entire US population and should not be generalized as such. Twitter may also reflect a loud minority of opinions, in particular strong opinions among those who opposed the policy. Further, this study identified tweets about Tobacco 21 within the months leading up to and the 11 days following enactment. A more in-depth analysis of tweets found that discussion about the law dropped off after December 31. For those interested in exploring discussion about Tobacco 21 in the future, it is important to note that Tobacco 21 Twitter chatter may change after implementation, and search terms employed to collect tweets related to Tobacco 21 will likely evolve over time due to changes in public conversation. For example, as champion of the first Tobacco 21 bill filed in April 2019, Mitch McConnell’s name was included within the search terms; however, searches about tweets related to Tobacco 21 after 2019 should not use his name to identify tweets relevant to this subject. Further, despite a careful coding procedure with multiple levels of redundancy, not all support or opposition may have been interpreted correctly due to sarcasm in tweets.

While the federal Tobacco 21 policy may have been a swift and appropriate regulatory action to address the e-cigarette epidemic and EVALI outbreak, it faces public opposition, especially in regard to enforcement among military personnel. Discussions of other age-related behaviors (e.g., military enlistment, alcohol use, legal voting age, and age to purchase a firearm) appeared to relate largely to anti-policy sentiment toward Tobacco 21. Thus, it is uncertain how well Tobacco 21 policies will be enforced within all communities in the US, particularly military members. It is important to note that the tobacco industry has historically supported negative policy components that decrease the efficacy of state tobacco control laws. Twitter discussions about the disjunction between other age-restricted behaviors and tobacco use may reflect the public’s view of Tobacco 21 on social media; however, it could alternatively be influenced by marketing messages created by the tobacco industry.

## Figures and Tables

**Table 1:** Operational Definitions and example tweets for each code.

Code	Operational Definition	Example Tweets
Relevance	The tweet mentions Tobacco 21 bill/law by including the age 21 and discussing the sale or purchase of tobacco and/or vaping products.	*FDA: It is now illegal to sell tobacco products to people younger than 21*
Federal Tobacco 21	The tweet mentions the federal Tobacco bill/law. The tweet does not include a state or local policy.	*These pesky butts are still clogging the swamp’s drain. Reaganesque opportunity to move T21 to all 50 states, and keep vaping flavors for adults available in specialty stores. # WeVapeWeVote for/against # Trump2020. Don’t be a Taft, support our small businesses*
Sentiment		
Pro-Policy	The tweet supports Tobacco 21.	*Only thing trumps done that I can agree with is him raising the legal age to purchase tobacco from 18 to 21*
Anti-Policy	If T21 is not supported/approved or described with sarcastic or negative contexts.	*I was just thinking, I’m not a smoker but I disagree with being 21 to buy tobacco. You are telling me that at 18 I can die in a war, but can’t smoke???*
Neutral-Policy	If the tweet is factual, but not opinionated or poses a question about unbiased facts/information about T21.	*why is changing the tobacco age in a spending bill?*
Type of Tobacco	The tweet mentions that it restricts access to…	
Cigarettes	cigarettes, cigarillos, or someone’s ability to smoke before 21 years.	*Fantastic. You’ve abridged the rights of adults, discriminated against them based on age. I’m so proud of you. If you’re an adult at age 18 for prison or military, you are an adult for alcohol and cigarettes.*
E-Cigarettes	e-cigarettes or vapor products.	*Congress Approves Raising Age to 21 for E-Cigarette and Tobacco Sales - The New York Times*
Nicotine	nicotine.	*I’ve said it all along … It should be 21 to buy cigs & ANY kind of nicotine*
Other	dip, cigars, hookah, or any other type of tobacco product.	*Anyone under 21 can no longer legally buy cigarettes, cigars or any other tobacco products in the U.S.*
Age-Restricted Behaviors	The tweet compares Tobacco 21 to…	
Enlisting in the Military	the age someone can enlist in the military.	*The only thing is they changed the age to buy smokes to 21 you can kill or be killed in war when 18 but you cant settle your nerves with a beer and a cig? thats B.S!!*
Voting	legal age to vote.	*You’re telling me at the age of 18 I’m allowed to vote, buy a house, car, credit card, and make the decision to put myself into years of debt. But I have to wait until I’m 21 to decide if I wanna drink, vape, or smoke? Sounds a bit ridiculous to me*
Purchase Firearm	the age someone can purchase a firearm.	*Mitch: We need to protect our youth from vaping!* *Also Mitch: Go ahead and die from guns, and if you’re 21 or older go ahead and die from vaping too!*
Alcohol	the age someone can purchase/consume alcohol.	*Can enlist in the military or ya know make porn yet, aren’t old enough to buy cigarettes or alcohol.*
Cannabis	the age someone can purchase/use cannabis.	*as someone who smokes tobacco i’m actually all for raising the smoking age to 21 because at least its consistent with alcohol and weed. smoking age was already 21 in ca but im all for consistent policy*
Adulthood	the age someone is considered an adult.	*People once you are 18 you can fight for your country, be tried as an adult … but cant buy cigs or alcohol.. why not just make the adult age is 21 and not 18.. and NO serving your country till 21..*
Other	other age-related behavioral restrictions.	*WAIT! i’m legally allowed to have my own house, get my own car insurance, go and play at the casino, AND get a loan at 18. but i’m no longer allowed BUY my own cigarettes??*

**Table 2:** Associations between Tobacco 21 Twitter Discussions and Policy Sentiment (N = 955).

		Pro		Anti		Neutral	
	N (%)	143 (15.0)	*p*	405 (42.4)	*p*	407 (42.6)	*p*
Type of Tobacco							
Cigarette/cigarillo	227 (23.8)	31 (13.7)	0.52	120 (52.9)	<0.001	76 (33.5)	<0.001
E-Cigarette/vapor product	193 (20.2)	42 (21.8)	0.003	55 (28.5)	<0.001	96 (49.7)	0.025
Nicotine	46 (4.8)	10 (21.7)	0.19	22 (47.8)	0.45	14 (30.4)	0.087
Age-Related Discussions							
Military Enlistment	170 (17.8)	15 (8.8)	0.013	140 (82.4)	<0.001	15 (8.8)	<0.001
Alcohol	103 (10.8)	17 (16.5)	0.65	69 (67.0)	<0.001	17 (16.5)	<0.001
Legal Voting Age	57 (6.0)	10 (17.5)	0.58	42 (73.7)	<0.001	5 (8.8)	<0.001
Purchase Firearm	36 (3.8)	8 (22.2)	0.21	23 (63.9)	0.008	5 (13.9)	<0.001
Cannabis	19 (2.0)	3 (15.8)	1.00 ^†^	8 (42.1)	0.98	8 (42.1)	0.96
Adulthood	35 (3.7)	3 (8.6)	0.28	30 (85.7)	<0.001	2 (5.7)	<0.001

^†^ Fishers exact test used to interpret *p*-value due to 25% of expected cell counts less than five.

## Data Availability

Data will be made available upon request.
